# Is membranous location of EGFR or EGFRvIII immunostaining associated with good prognosis in renal cell carcinoma?

**DOI:** 10.1038/sj.bjc.6602843

**Published:** 2005-10-18

**Authors:** H Modjtahedi, M P Cunningham

**Affiliations:** 1 Division of Oncology, Postgraduate Medical School, University of Surrey, Guildford GU2 7WG, UK

**Sir**,

We read with great interest the published article by Kallio *et al* (2003) in the British Journal of Cancer on the association between the location of EGFR immunostaining and overall survival in renal cell carcinoma (RCC) patients. In this paper, the authors have reported that overall survival was significantly longer (*P*=0.004) in patients with prominent membranous EGFR expression compared to patients with either EGFR-negative tumours or tumours with predominantly cytoplasmic EGFR staining (Kallio *et al*, 2003). This is an important finding, as the expression of membranous EGFR has often been associated with a poor prognosis in cancer patients (Lager *et al*, 1994; Moch *et al*, 1997), while other studies have found either no association between EGFR expression and prognosis (Hofmockel *et al*, 1997) or more recently an association between the expression of cytoplasmic EGFR and poor prognosis in RCC patients (Langner *et al*, 2004).

To our knowledge, the paper by Kallio *et al* is the first paper to describe an association between prominent membranous EGFR immunostaining and longer overall survival in RCC patients. However, there is a conflicting statement in the Materials and Methods section of the paper by Kallio *et al* that prevents us from accepting their conclusion and the authors need to clarify/rectify accordingly. In the immunohistochemical staining section of the Materials and Methods, Kallio *et al* stated the use of a polyclonal rabbit anti-EGFR variant III antibody (EGFRvIII) for EGFR immunostaining. The EGFRvIII is a ligand-independent, constitutively active and mutated form of EGFR (Pederson *et al*, 2001). Did the author use the rabbit anti-EGFRvIII antibody in their study and if so does it crossreact with the EGFR? Could Kallio *et al* clarify/rectify whether the prominent membranous EGFRvIII immunostaining in that study was associated with a good prognosis in patients with RCC? While no clear association has been found between the expression of the EGFR and response to the EGFR inhibitors in cancer patients, including patients with RCC, the expression of membranous EGFR and/or EGFRvIII in RCC patients would, however, make them an ideal target for therapy with the anti-EGFR antibodies (Modjtahedi *et al*, 2003; Dawson *et al*, 2004; Rowinsky *et al*, 2004; Dancey, 2004). We would appreciate clarification from Kallio *et al*.
